# Can immunohistochemical biomarkers distinguish epithelial dysplasia degrees in actinic cheilitis? A systematic review and meta-analysis

**DOI:** 10.4317/medoral.23223

**Published:** 2019-12-24

**Authors:** Thalita Santana, Bruno Matuck, Jefferson R. Tenório, Mariana Minatel Braga

**Affiliations:** 1 PhD student at School of Dentistry, Department of Oral Pathology, University of São Paulo; 2 MSc at School of Dentistry, Department of Oral Pathology, University of São Paulo; 3 PhD and Professor at Department of Pediatric Dentistry, School of Dentistry, University of São Paulo, São Paulo, Brazil

## Abstract

**Background:**

Actinic cheilitis (AC) is a poten-tially malignant disorder of the lip, characterized by epithelial and connective tissue alterations caused by chronic exposure to ultraviolet radiation. In the past decades, diverse studies have been conducted in lip carcinogenesis and many biomarkers have been identified in lip lesions, yet there is no scientific evidence that determines its usefulness in the clinical setting or in histopatho-logical routine. Therefore, we conducted the first systematic review in this field to summarize the results of published studies on immunohistochemical bi-omarkers in lip carcinogenesis, to evaluate if there is a marker than can distin-guish the different histological grades of AC.

**Material and Methods:**

Retrospective stud-ies that investigated immunohistochemical biomarkers in AC defined on standardised histological assessment were gathered from five databases and evaluated. Each study was qualitatively evaluated using the Critical Appraisal Tools from SUMARI.

**Results:**

The proliferation marker Ki-67 was the most studied biomarker and we observed, through meta-analysis, that it was differently expressed between AC and lip cancer, but not in AC sub-groups. Most articles had a high risk of bias.

**Conclusions:**

In summary, the literature lacks quality follow up studies in actinic cheilitis. Multi-centre cohort studies, with patients stratified by treatment type and the use of image analysis soft-ware, could be the solution to further address the issues of investigating poten-tially malignant lesions and help change clinical practice, in terms of individu-alizing patients’ treatment and prognosis prediction.

** Key words:**Lip carcinogenesis, actinic cheilitis, lip cancer, bi-omarkers.

## Introduction

Lip squamous cell carcinoma (LSCC) represents 20-30% of all oral cavity tumors and it deserves a specific attention, especially in its pathogeny, that differs from oral squamous cell carcinoma (OSCC) (1). OSCC is related to chronic consumption of alcohol and tobacco, while LSCC is closely related to chronic exposure to the ultraviolet (UV) radiation of sun (1). The establishment of LSCC is preceded by clinical and histological alterations in the lip, which is known as actinic cheilitis (AC). AC is re-garded as a potentially malignant lesion and it is characterized as a degenerative disorder, affecting mainly white males over 40-years-old, that usually work outdoors (2). Histologically, this lesion is characterized by cyto-logical and architectural modifications, epithelial dysplasia and solar elastosis (basophilic degeneration of elastic fibres) (3).

To facilitate patient management, grading systems for oral epithelial dysplasia have been proposed. According to the WHO (4), epithelial dysplasia can be characterized as mild, moderate or intense, according to cyto-logical and architectural alterations. However, this system cannot predict patient’s progno-sis and is regarded by pathologists as subjective (5). In 2006, Kujan *et al*. (6) proposed a binary graduation system for oral dysplasia, in order to minimize analysis subjectivity. This new system preconizes the division of the lesions in two subgroups, according to the risk of malignant transformation (low risk and high risk).

In the past years, researches have tried to elucidate the mechanisms underlying oral epithelial dysplasia. Thereby, many different immunohistochemical biomarkers have been investigated in oral carcinogenesis and a compilation of these results has been outputted (7); yet, to our best knowledge, there are no systematic reviews on biomarkers of lip carcinogenesis, and researchers and practioners are still not able to determine which AC cases will undergo malignant transfor-mation.

For this reason, we conducted a systematic review to examine if there is some im-munohistochemical biomarker that could be related to the degree of epithelial dysplasia in AC.

## Material and Methods

This systematic review was reported according to the Preferred Reporting Items for Systematic Reviews and Meta-Analyses PRISMA Checklist (available at: http://www.prisma-statement.org). The review protocol was registered at the International Prospective Register of Systematic Reviews (PROSPERO) under number CRD 42017055294.

- Study design

We conducted a systematic review of human studies to summarize the results of pub-lished studies on immunohistochemical biomarkers in lip carcinogenesis, in order to eval-uate if there is a marker than can distinguish the different histological grades of actinic cheilitis (AC).

- Search strategy

We searched and identified articles of the following bibliographic databases: Pub-Med, Scopus, Web of Science, ScienceDirect and Scielo. The search included all articles published up to April 25th, 2017, with no time restrictions. Duplicated references were excluded by a reference manager software (Mendeley Desktop version 1.17.9).

The search strategy used for PubMed was: ((((((actinic cheilitis[Title/Abstract]) OR actinic cheilosis[Title/Abstract]) OR solar cheilosis[Title/Abstract]) OR solar cheilitis[Title/Abstract]) OR lip carcinogenesis[Title/Abstract]) OR lip photocarcinogene-sis[Title/Abstract]) AND immunohistochem*[Title/Abstract]. For the other databases we conducted independent searches using the first block: (actinic cheilitis OR actinic cheilosis OR solar cheilosis OR solar cheilitis OR lip carcinogenesis OR lip photocarcinogenesis), in order to maximize the inclusion of relevant studies.

- Inclusion criteria

Prospective and retrospective studies that investigated immunohistochemical bi-omarkers in AC defined on standardised histological assessment as outlined by the WHO (4) and/or Kujan (6).

- Exclusion criteria

The following exclusion criteria were applied: (a) Scientific papers that did not report AC histological grading; (b) Lack of comparison between biomarkers among AC groups or between AC and control (normal lip mucosa or lip squamous cell carcinoma); (c) Stud-ies that investigated immunohistochemical biomarkers in samples other than paraffinized material; (d) Reviews, single case reports, clinical trials, letters, personal opinions, book chapters, and conference abstracts.

- Study selection

The study selection was conducted by two authors (BM and TS), who independently reviewed the titles and abstracts of all the papers, and selected the studies that met the in-clusion criteria. A kappa test was performed to verify agreement between authors and we obtained a reliable result of 0.87. Afterwards, both authors independently evaluated all full articles to determine if they reported the expression of immunohistochemical biomarkers in the subgroups of AC, based on histological grading (kappa score = 1). If there were any disagreements between the authors, they were resolved by mutual consensus. Final selection was always based on the full-text of the publication.

- Data collection

Two authors (BM and TS) collected the information from the included papers. The following information was gathered and presented in Tables: study characteristics (author, year of publication, country); population (sample size, cases of AC, LSCC and normal lip controls); type of histological grading performed; immunohistochemical biomarkers that were analysed; expression of biomarkers in each subgroup (AC, LSCC and control); sta-tistical tests performed; main conclusions. A partial grey literature search was performed using Google Scholar in order to investigate detailed results from PhD and Master’s de-gree thesis and dissertations, and perform the meta-analysis.

- Meta-analysis 

Due to high heterogeneity in immunopositive cells counting/scoring for the studied biomarkers and contrasting results presentation in the articles, we included only the pro-tein Ki-67 for the meta-analysis. This was one of the most studied proteins and the only one that had a standardized analysis among the studies. To be included for meta-analysis, the articles (or their respective thesis/dissertations) had to report the mean and standard deviation of Ki-67 immunopositive cells in each of the following groups: control, mild dysplasia AC, moderate dysplasia AC, severe dysplasia AC, low grade LSCC, moder-ate/high grade LSCC. We used a Mixed-effects Model to estimate the amount of residual heterogeneity (tau2) and unaccounted variability (I2) among groups. The analysis was per-formed with the R software, package metafor 1.9-8.

- Risk of bias in individual studies

Each selected study was qualitatively evaluated using the Critical Appraisal Tools from SUMARI (System for the Unified Management, Assessment and Review of Infor-mation), proposed by the Joana Briggs Institute (available at: http://joannabriggs.org/research/critical-appraisal-tools.html). Since almost all cases com-prised retrospective studies, with samples chosen by convenience and lack of follow-up, we used the Critical Appraisal Tool for Case Series. This type of study is described as the kind in which “only patients with the outcome are sampled (either those who have an ex-posure or those who are selected without regard to exposure), which does not permit cal-culation of an absolute risk” (8). In our case, the exposure is the lesion actinic cheilitis. The evaluated items were scored “Yes”, “No” or “Unclear” for each paper individually (Table 1).

**Table 1:**
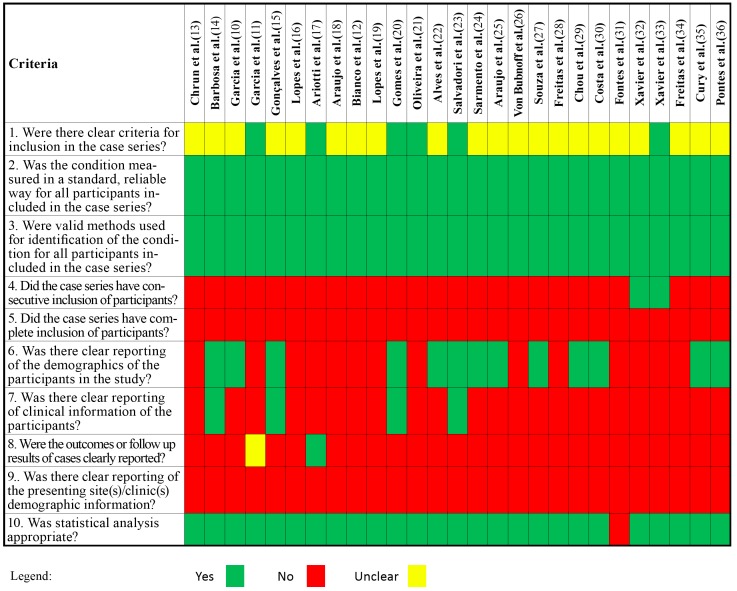
Risk of bias of selected studies according to the Critical Appraisal Tool for Case Reports (SUMARI).

## Results

- Study selection

**Figure 1:**
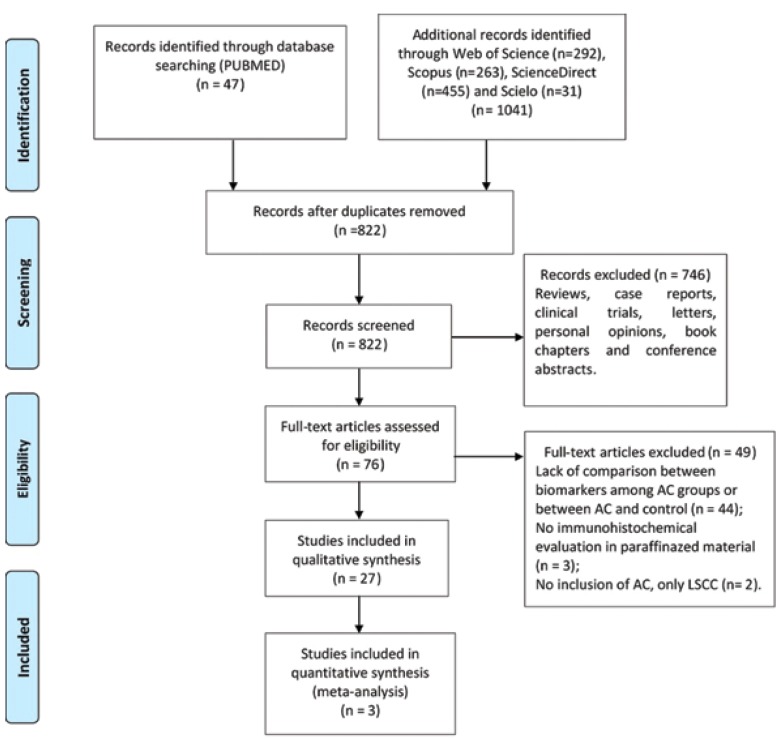
Flow diagram for study selection.

1088 articles were identified across the five electronic databases. After removing the duplicates, 822 articles remained. A comprehensive evaluation of the titles and abstracts resulted in the exclusion of 746 articles, with the remaining 76 articles being allocated to full text in-depth review. This process led to the exclusion of 49 studies. Finally, 27 arti-cles were retained for qualitative analysis and three articles were selected for meta-analysis. A flow chart detailing the process of identification, inclusion, and exclusion of the studies is shown in Fig. 1.

- Study characteristics 

All reviewed articles comprised retrospective studies (10-36) (Table 2). The studies presented a great geographic polarization; from the 27 analyzed articles, 25 where originated from Brazil, one from Germany and one from the USA. The included studies were published between 2003 and 2017. The number of AC cases in each study varied from 10 to 70, while LSCC cases went from 0 to 65 cases. Forty-one [41] different proteins were researched in these arti-cles, and the relation between these proteins expressions among LSCCs and ACs was investigated in 20 papers. The mean number of studied ACs was 34.5 cases per article, while the mean of LSCC was 32 cases. Only five articles used the binary grading system proposed by Kujan *et al*. (Table 1).

**Figure 2:**
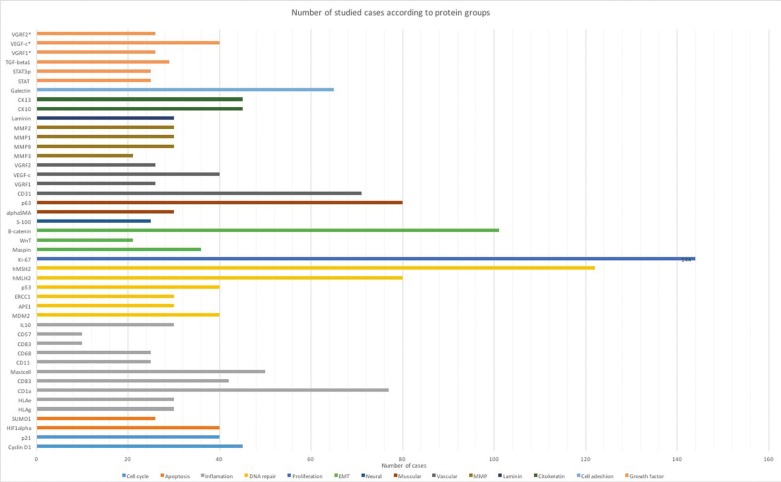
Number of studied cases according to protein groups.

Among the studied biomarkers, the ones that were most investigated were DNA re-pair proteins, with 12 antibodies assessed. The inflammatory markers were the second most assessed group. Other groups of proteins were also analyzed, including apoptosis markers, metalloproteins, cell cycle markers, growth factors, neural and muscle markers (Fig. 2). This variety of biomarkers hampered any analyses between the articles.

**Table 2:**
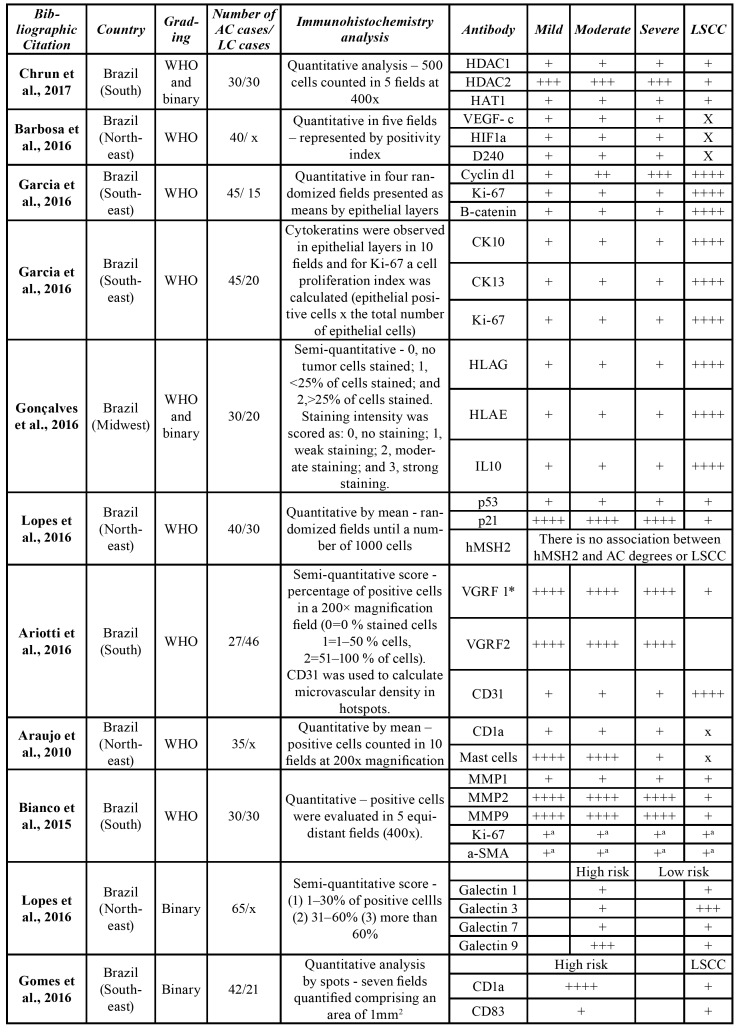
Details of selected studies.

**Table 2 cont.**
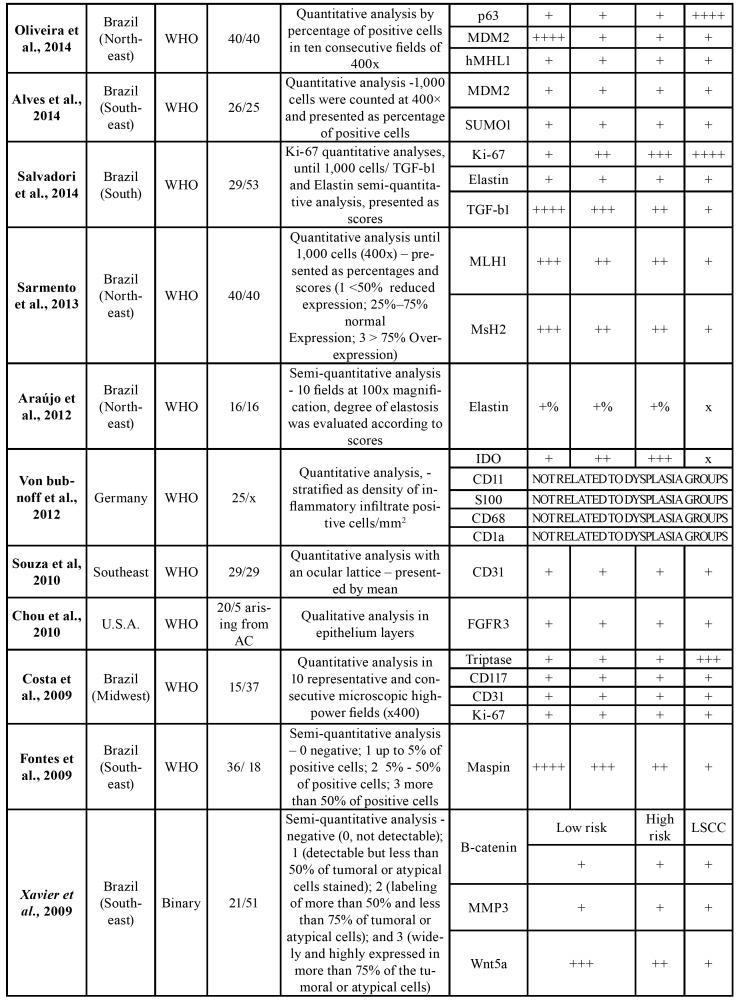
Details of selected studies.

**Table 2 cont.**
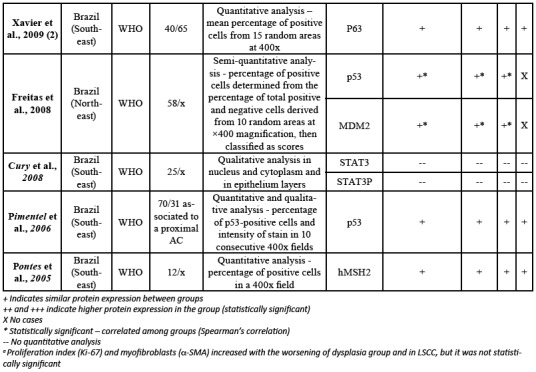
Details of selected studies.

- Risk of bias

After analyzing all included studies with SUMARI critical appraisal tool, we ob-served that most articles (n=22) had unclear criteria for inclusion of the cases. We consid-ered a clear inclusion criterion when the study reported when the cases were diagnosed, where they were collected and what were the sample inclusion/exclusion criteria (even if it was a convenience sample). Also, only three studies reported to have consecutive inclu-sion of cases. However, all studies reported a reliable measurement of AC degrees of dysplasia according to the grading systems proposed by WHO (4) and Kujan *et al* (6).

Thirteen articles reported the patients’ demographics, while clinical information was available in only four of the reviewed studies. Information regarding the patient’s out-come/follow-up was available in only one study (9) and partially available in another one (10).

Almost all studies reported to use clear statistical methods to compare the variables, however they were very diverse, since the researchers applied different methodologies for cell counting, with no clear cutoff of what was positive/negative or low/high. Also, maybe due to limited number of cases, authors tended to group AC cases for statistical analysis, and this grouping was very heterogeneous among studies.

- Meta-analysis

**Figure 3:**
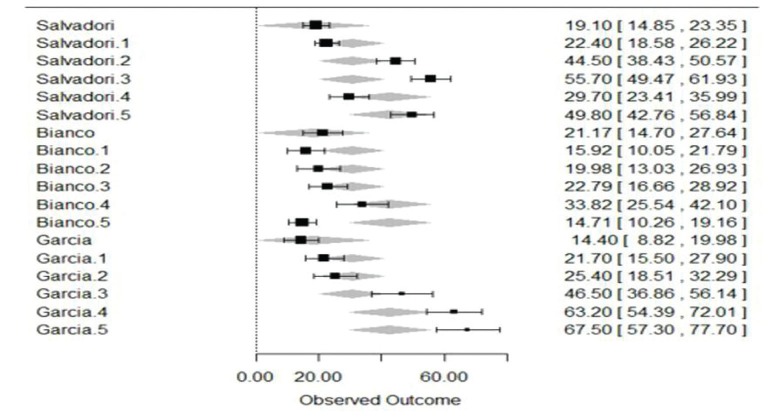
Forest plot showing the differences in Ki-67 expression among control (no number), AC with mild dyspla-sia (.1), AC with moderate dysplasia (.2), AC with severe dysplasia (.3), low grade LSCC (.4) and moderate/high grade LSCC (.5).

Three studies were selected for meta-analysis (11,12,23). We compared the mean expression of Ki-67 among the groups of AC, LSCC and control and observed a high heterogeneity among the studies (tau2=241.02; I2=95.91%). We observed that Ki-67 mean expression was similar in control groups and was higher in LSCC than in AC. However, it varied re-markably among AC subgroups. This information is summarized in a forest plot in Fig. 3.

## Discussion

One of the main purposes of investigating immunohistochemical biomarkers in lip lesions is observing if there are differences between protein expressions in different grades of AC or between AC and LSCC or control/normal lip. Usually, this is done not for diagnostic purposes, but with the expectation to develop future prognostic markers, which could possibly set apart cases that will undergo malignant transformation.

In the past two decades a significant number of studies have been conducted in lip carcinogenesis and many biomarkers have been identified in lip lesions, yet researchers are still not able to determine its usefulness in the clinical setting or in histopathological routine (37). The histological grading sys-tem proposed by WHO (4) for epithelial dysplasia in AC is extensively used by oral pathologists, anyhow, clinical experience has shown that even cases of mild oral dysplasia can develop into LSCC (38). In 2006, Kujan *et al*. (6) proposed a new binary grading system for AC, however a decade later few studies applied this system for histological assessment (5,39).

In this review, only few papers investigated the same biomarkers, which made it im-practical to make comparisons between studies. The proliferation marker Ki-67 was the most studied biomarker, anyhow only three papers studying this protein met the inclusion criteria for meta-analysis. We observed that Ki-67 is differently expressed among AC and control and between AC and LSCC, however its expression was highly variable among AC groups.

Despite we have identified many studies in this review, almost all of them comprise case series, with cross-sectional analyses, lacking quality follow-up data for AC cases and are therefore unsuiTable to in use in prognosis analysis. We recognize that this is one of the main issues of studying oral potentially malignant disorders, since they may or not undergo malignant transformation in an unknown period of time, and it is challenging to follow-up patients continuously.

Also, most reviewed studies failed to specify the inclusion criteria for studied cases and samples were chosen by convenience, which is a potential source of bias and it can reduce the level of evidence of the studies. Besides, many articles did not report important clinic and demographic data of the patients, nor did they report the presenting site’s clin-ic/demographic information. Therefore, it is not possible to make comparisons between patient’s characteristics and expression of immunohistochemical biomarkers. Further-more, the literature is inconsistent regarding the evaluation of biomarkers positivity or low/high expression, since researchers uses different quantitative or semi-quantitative methodologies for cell evaluation, which makes it difficult to compare studies with the same biomarkers.

Notwithstanding, another possible source of bias is the statistical analysis performed for each study. Even though most authors statistically analysed their results, we observed a tendency in grouping AC subgroups (e.g. all cases of AC independently of dysplasia grading) and comparing it only to LSCC or control, to achieve statistically significant re-sults. This could be due to small sample sizes, as it could possibly be related to selective reporting bias, which may happen as a result of the belief that scientific journals will not to accept papers reporting only “negative” results (not statistically significant). Additional-ly, nearly all evaluated studies are not replicable or reproducible, since important data are often not reported. According to Peng [2015] (40), there are two major components to a reproducible study: that the raw data from the experiment are available; and that the statistical code and documenta-tion to reproduce the analysis are also available.

At last, we acknowledge another risk of bias within this review, since almost all in-cluded studies are from Brazilian research groups. This can be explained by the fact that in tropical countries, rural workers are chronically exposed to high levels of solar radiation throughout the year, which explains an AC prevalence of up to 28.4% in Brazilian popu-lations (41). Considering that, a great number of studies in this field are conducted in this country. Also, we have thoroughly analysed all published studies in AC and although there are papers from USA, Chile, Australia, Greece, Spain and Germany, for example, only two of them met the inclusion criteria in this review. The other researches that investigated biomarkers in AC did not report comparisons between epithelial dysplasia groups and therefore were excluded.

As regards the meta-analysis, we also acknowledge its limitation. Since only three articles could be included for statistical analysis, we observed high heterogeneity among results, specially between AC groups, and this may not represent the reality of Ki-67 staining in AC or LSCC. One of the studies that investigated this protein (10) had to be excluded from meta-analysis since we believe the authors analysed the same sample used in their previous study (11), which was included.

- Implications for research and practice

In this review, we have identified 76 studies that investigated biomarkers in the field of lip carcinogenesis. Despite this significant number, well documented cohort studies are still limited. We still ought to understand the behaviour of AC and its progression to can-cer, in order to apply it clinically. We emphasize the difficulty in accessing complete fol-low-up data and highlight the need for further clinical research in potentially malignant disorders. As suggested in a systematic review by Smith *et al*. (7), multi-centre cohort studies, with patients strati-fied by treatment type, could be the solution to further address the issues of investigating those lesions.

We recommend that in studies of biomarkers of lip carcinogenesis histological grad-ing is performed for AC and LSCC, preferentially using more than one grading system. Also, comparisons with normal epithelium are indispensable. Thoroughly describing the methodology used for quantifying the antibodies is crucial for reproducibility of the study and, ideally, a unified methodology should be adopted. Maybe with the aid of an image software, to reduce examiners’ observation variability, this could be achieved. Likewise, results should be described more carefully, with Tables showing the results for each AC and LSCC subgroup, as well as control groups. Clinic and demographic information are also important to be described.

## Conclusions

We observed that the different studied proteins are similarly expressed in AC epithe-lial dysplasia grades, therefore are not useful in differentiating them. However, the poten-tial use of some biomarkers to differentiate AC and LSCC has been demonstrated. We believe that soon some of them could become useful in identifying cancer risk in patients with actinic cheilitis. If we can develop reliable and reproducible follow-up studies, we will be able to change clinical practice in terms of individualizing patients’ treatment and prognosis prediction. Clearly, further research is needed to exploit the many possibilities in lip carcinogenesis.
